# Ontogeny of symbiont community structure in two carotenoid‐rich, viviparous marine sponges: comparison of microbiomes and analysis of culturable pigmented heterotrophic bacteria

**DOI:** 10.1111/1758-2229.12739

**Published:** 2019-03-06

**Authors:** Oriol Sacristán‐Soriano, Marina Winkler, Patrick Erwin, Jeremy Weisz, Olivia Harriott, Gary Heussler, Emily Bauer, Brittany West Marsden, April Hill, Malcolm Hill

**Affiliations:** ^1^ Department of Biology University of Richmond Richmond VA USA; ^2^ Marine Ecology Department Centro de Estudios Avanzados de Blanes (CEAB, CSIC) Blanes Spain; ^3^ Department of Biology and Marine Biology, Center for Marine Science University of North Carolina Wilmington NC USA; ^4^ Department of Biology Linfield College McMinnville OR USA; ^5^ Department of Biology Fairfield University Fairfield CT USA

## Abstract

Marine sponges harbour diverse communities of microbes. Mechanisms used to establish microbial symbioses in sponges are poorly understood, and the relative contributions of horizontal and vertical transmission are unknown for most species. We examined microbial communities in adults and larvae of carotenoid‐rich *Clathria prolifera* and *Halichondria bowerbanki* from the mid‐Atlantic region of the eastern United States. We sequenced microbiomes from larvae and their mothers and seawater (16S rRNA gene sequencing), and compared microbial community characteristics between species and ambient seawater. The microbial communities in sponges were significantly different than those found in seawater, and each species harboured a distinctive microbiome. Larval microbiomes exhibited significantly lower richness compared with adults, with both sponges appearing to transfer to larvae a particular subset of the adult microbiome. We also surveyed culturable bacteria isolated from larvae of both species. Due to conspicuous coloration of adults and larvae, we focused on pigmented heterotrophic bacteria. We found that the densities of bacteria, in terms of colony‐forming units and pigmented heterotrophic bacteria, were higher in larvae than in seawater. We identified a common mode of transmission (vertical and horizontal) of microbes in both sponges that might differ between species.

## Introduction

Sponges are emblematic of symbiotic consortia involving animals and microbes (Erwin *et al*. 2015; Thomas *et al*. 2016; Hill and Sacristán‐Soriano 2017; Moitinho‐Silva *et al*. 2017a). Sponge: microbial symbioses are found in all marine habitats, and involve a taxonomically‐diverse array of microorganisms (Maldonado *et al*. 2005; Caporaso *et al*. 2011; Schmitt *et al*. 2011; Taylor *et al*. 2007, 2013; Thomas *et al*. 2016; Moitinho‐Silva *et al*. 2017a). Equally diverse types of ecological associations occur among these partners. The associations can range from facultative to obligate, and sponge symbionts occur intracellularly, intercellularly and epizoically (Simpson 1984; Taylor *et al*. 2007; Hill and Sacristán‐Soriano 2017). Despite their ubiquity, we have a limited understanding of how bacterial communities are initiated in sponge hosts, or the forces that shape the ecological structure of these microbiomes. The composition of microbial communities in sponges is generally host species‐specific, and not a random sample of microbes from the environment (e.g., Fieseler *et al*. 2004; Hill *et al*. 2006; Taylor *et al*. 2007; Isaacs *et al*. 2009; Radwan *et al*. 2010; Gerçe *et al*. 2011; Schmitt *et al*. 2011; Erwin *et al*. 2012a,b, 2015; Pita *et al*. 2013; Burgsdorf *et al*. 2014; Sipkema *et al*. 2015; Steinert *et al*. 2016; Hill and Sacristán‐Soriano 2017). Indeed, the same sponge species sampled from distinct geographic regions at different times harbour remarkably uniform bacterial communities (Hentschel *et al*. 2002, 2006; Montalvo and Hill 2011; Burgsdorf *et al*. 2014). However, 77 of the 173 previously described ‘sponge‐specific’ clusters have been detected in seawater (Taylor *et al*. 2013). Furthermore, as filter‐feeding bacteriotrophs, adult sponge bodies are filled with environmentally‐derived water, which introduces non‐trivial challenges in identifying true sponge‐associates from transient food items (e.g., Enticknap *et al*. 2006; Schmitt *et al*. 2007; Sharp *et al*. 2007; Schmitt *et al*. 2011).

The goal of the research presented here was to compare microbiomes found in adult and larval sponge tissues. Our objective was to explore aspects of the ontological development of these bacterial communities to gain insights into the relative importance of direct (i.e., vertical, maternal or ‘closed’) and indirect (i.e., horizontal, environmental or ‘open’) routes of transmission (Funkhouser and Bordenstein 2013; Douglas 2015). While theory predicts that vertical transmission should favour mutualist symbionts due to coupling of host and symbiont interest, horizontal transmission is a common strategy in many mutualistic symbioses (Hartmann *et al*. 2017). Hartmann *et al*. (2017) proposed that horizontal transmission may mitigate conflicts‐of‐interest between symbiont transmission rates and the health of early reproductive stages given that selection might favour bacterial transmission into eggs/embryos whether or not that transmission damaged host reproduction. In contrast, empirical evidence in jellyfish:*Symbiodinium* partnerships indicates that horizontal transmission can lead to exploitative symbioses with a breakdown in mutualism (Sachs and Wilcox 2006). Thus, the evolutionary processes that generate these transmission modes are not well understood for symbionts or hosts (Sachs 2015; Hartmann *et al*. 2017). In addition, ecological rules of entry into, and subsequent development of, symbiont microbial communities (open or closed) are poorly understood. For example, community characteristics within the host (e.g., connectivity) are likely influenced to a greater or lesser extent by mode of transmission ‐ it is clear that additional work is required in this area.

Studies focused on transmission of sponge symbionts between generations have used three main methods: microscopy, molecular analysis (i.e., DNA sequencing) and culturing of bacterial symbionts. Vacelet (1975) was among the first to demonstrate that vertical transmission in sponge: microbe symbioses was possible in oviparous sponges (see also Gallissian and Vacelet 1976; Lévi and Lévi 1976). Since that time, several microscopic studies have demonstrated vertical transmission of bacterial symbionts through sponge eggs and larvae (Gaino *et al*. 1987; Sciscioli *et al*. 1989, 1991, 1994; Kaye 1991; Gaino and Sara 1994; Usher *et al*. 2001, 2005; Ereskovsky *et al*. 2005; Maldonado 2007; Schmitt *et al*. 2007; De Caralt *et al*. 2007). Indeed, some sponges appear to have specialized morphological structures involved in symbiont transition (i.e., the ‘umbilici’ of Kaye 1991). Several studies have used molecular approaches to explore intergenerational transmission of symbionts (Oren *et al*. 2005; Schmitt *et al*. 2007; Sharp *et al*. 2007; Steger *et al*. 2008; Lee *et al*. 2009; Gloeckner *et al*. 2013; Sipkema *et al*. 2015). Schmitt *et al*. (2008) examined microbial symbionts transmitted vertically in eight sponge species representing different modes of reproduction and having distinct low microbial abundance (LMA)/high microbial abundance (HMA) status. They identified 28 vertical‐transmission clusters, defined as clades including microbes found in adults and their offspring (see also Webster and Blackall 2009). Enticknap *et al*. (2006) used a culture‐based approach to study an α‐proteobacterium isolated from eight sponge species from the Caribbean and Indo‐Pacific (see also Selvin *et al*. 2009). While these approaches offer insight into the holobiont, the integration of culture‐dependent and culture‐independent techniques can provide a more complete picture of the host:symbiont relationships.

In the present study, we examined microbial communities in adults and larvae of *Clathria prolifera* and *Halichondria bowerbanki* from the Chesapeake Bay to gain a deeper knowledge of modes of transmission of microbes between generations. We focused on these sponges due to their bright pigmentation [red in *C. prolifera* (i.e., Red Beard Sponge), and yellow in *H. bowerbanki* (i.e., Yellow Sun Sponge)]. Carotenoids have been isolated from many marine sponges (e.g., Eimhjellen 1967; Parisi *et al*. 1977; Tanaka *et al*. 1977; Litchfield and Liaaen‐Jensen 1980; Liaaen‐Jensen *et al*. 1982; Simpson 1984; Lee and Gilchrist 1985; Sliwka *et al*. 1987; Hooper *et al*. 1992), and may be acquired from symbionts or diet (Liaaen‐Jensen 1967; Miki *et al*. 1994). The conspicuous coloration in *C. prolifera* and *H. bowerbanki* is also present in the viviparous larvae of both species (see also Lindquist and Hay 1996). We used a culture‐independent characterization of microbial communities found in larvae, mothers and surrounding seawater using partial (V4 region) 16S rRNA gene sequences. To avoid the trophic‐generated, environmental noise in the prokaryotic communities associated with sponges, and to maximize the probability of examining true sponge microbial associates, we focused on the non‐feeding lecithotrophic larvae of both species. We also used a culture‐based survey to identify pigmented bacteria in both sponge hosts, and characterized selected functional traits from these isolates to provide a more complete picture of the relationships through potential linkages between symbiont and host phenotype.

## Results

### *Microbiome characterization*

The V4 region of the 16S rRNA gene was sequenced on an Illumina MiSeq platform (Supporting Information File S1) and a total of 2432776 reads were obtained after denoising and quality filtering with a library depth ranging from 57348 to 145713 reads. To avoid artefacts of varied sampling depth, we rarefied our libraries to the lowest read count (*n* = 57348; Supporting Information Fig. S1). Thirty nine bacterial and four archaeal phyla were detected in the 6677 OTUs recovered from seawater, *Clathria prolifera* and *Halichondria bowerbanki* samples. Of these, 1779 OTUs were unique to *H. bowerbanki*, 1656 OTUs were unique to *C. prolifera* and 500 OTUs were found in both sponge species but not seawater. Seawater exhibited fewer unique OTUs (*n* = 982) but shared 943 OTUs with both sponge species (Supporting Information Fig. S2).

**Figure 1:**
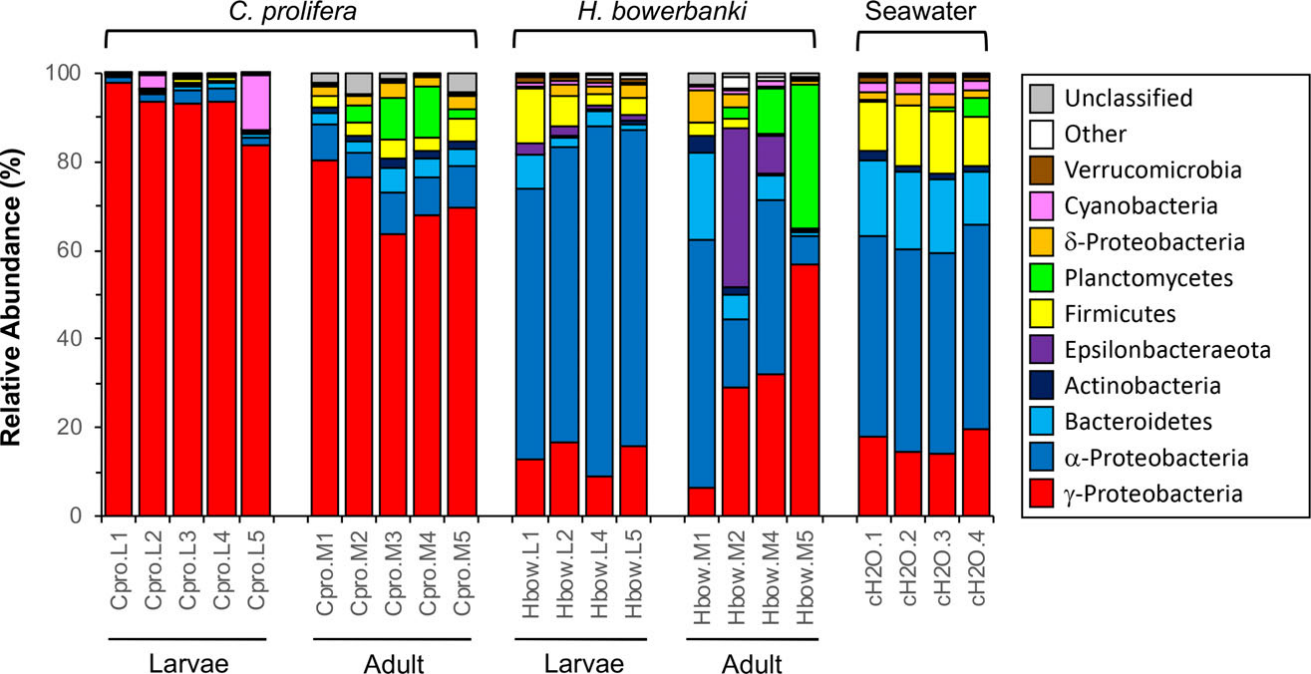
Taxonomic composition of bacterial communities in healthy *Clathria prolifera* (Ellis and Solander 1876) larvae (*n* = 5; 50 pooled larvae each) and adults (*n* = 5), *Halichondria bowerbanki* (Burton 1930) larvae (*n* = 4; 50 pooled larvae each) and adults (*n* = 4) and ambient seawater (*n* = 4). Sponges were collected from 0.5 to 1 m below the mean low water mark on pier pilings off of Gloucester Point, Virginia (USA; 37.24759, −76.49971), the same day during the late spring/early summer 2017.

The taxonomic composition of microbial communities recovered from ambient seawater, and from *C. prolifera* and *H. bowerbanki* sponge hosts, were significantly different (Fig. 1). The microbial community harboured by *C. prolifera* was enriched for γ‐Proteobacteria (>80% of the microbial community, on average) compared with seawater (<17% of the community) and *H. bowerbanki* (<23%). However, microbial communities in *C. prolifera* were depleted in members of α‐Proteobacteria (<6%) compared with seawater (>45%), especially for the larvae (<3%). *Clathria prolifera* larvae had reduced Bacteroidetes, Actinobacteria, Firmicutes, Planctomycetes and δ‐Proteobacteria populations compared with their mothers. Some *C. prolifera* larvae had slightly more variable populations of Cyanobacteria than their mothers. *Halichondria bowerbanki* exhibited greater interindividual and intergenerational variability in the taxonomic composition of their microbiome (Fig. 1). Compared with ambient seawater, some individuals were enriched for members of the γ‐Proteobacteria, Epsilonbacteraeota and Planctomycetes, other individuals were depleted in terms of proportional representation of the Bacteroidetes, Actinobacteria and Firmicutes. *H. bowerbanki* larvae had a significantly enriched microbiome community in α‐Proteobacteria compared with *C. prolifera*, and, most notably, their mothers (one‐way ANOVA, *P* < 0.001; Fig. 1). Unlike their mothers, *H. bowerbanki* larvae harboured barely detectable populations of Planctomycetes (<0.1%), and were slightly enriched for Firmicutes.

### *Community‐level analysis*

**Figure 2:**
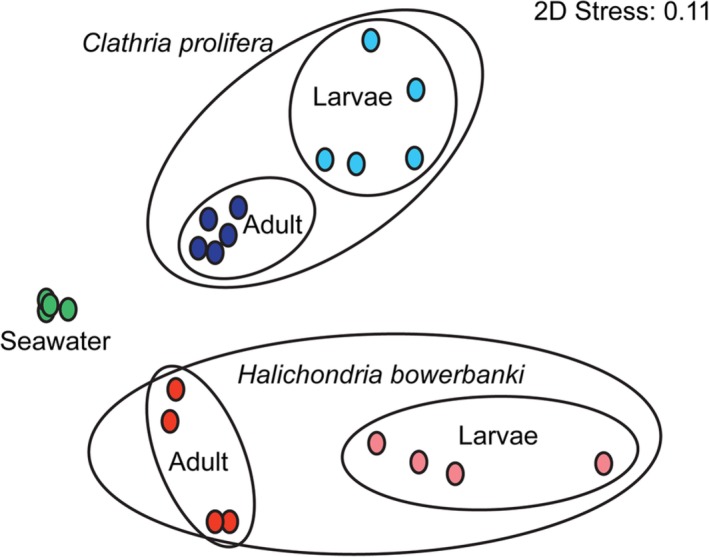
Nonmetric multidimensional scaling plot of bacterial community structure from replicate individuals of *Clathria prolifera* (dark blue ‐ mother sponges, light blue ‐ larvae), *Halichondria bowerbanki* (red ‐ mother sponges, pink ‐ larvae) and seawater (green). Plots based on square‐root transformed, Bray–Curtis similarity matrices were constructed in Primer (version 7.0.13) from next‐generation OTU relative abundance data. Stress value for two‐dimensional ordination is shown.

Statistically significant differences in community structure (PERMANOVA) were detected among *C. prolifera*, *H. bowerbanki* and seawater microbiomes (*F*_2,19_ = 5.682; *P* = 0.001; Fig. 2). The source of microbial samples explained >45% of the variation in community structure (PERMANOVA) and samples clustered depending on whether they were isolated from seawater, *C. prolifera* or *H. bowerbanki* (Fig. 2). In addition, a significant interaction between host species and life stage occurred among sponge samples (PERMANOVA, *F*_1,17_ = 2.579; *P* = 0.009), with significant pairwise differences in community structure detected between larvae and adults of *Clathria prolifera* (*t* = 2.161, *P* = 0.009) and *Halichondria bowerbanki* (*t* = 1.810, *P* = 0.035; Supporting Information File S1 for details).

Multivariate dispersion analysis revealed higher variability within *H. bowerbanki* microbial communities compared with *C. prolifera* communities from adult/maternal tissue (*t* = 3.914; *P* = 0.006; Fig. 2), but microbiomes in larval tissue of these two species were equally variable (*t* = 0.781; *P* = 0.684). Microbial communities from *C. prolifera* were more variable in larvae than they were in maternal tissue (*t* = 5.504; *P* = 0.006), but *H. bowerbanki* microbial communities were equally variable regardless of developmental stage (*t* = 0.263; *P* = 0.839; Supporting Information File S1).

We observed differences in mean values of richness, diversity (i.e., inverse Simpson diversity index) and evenness in symbiont communities between host species and life stages (Table 1; see Supporting Information File S1 for details). A two‐way ANOVA detected a significant interaction term for species richness (*F*_1,14_ = 6.397; *P* = 0.024), and pairwise comparisons indicated that adults harboured statistically richer microbial communities than larvae in both species (*C. prolifera*: *q* = 8.544; *P* < 0.001 and *H. bowerbanki*: *q* = 12.441; *P* < 0.001). Microbial communities in *H. bowerbanki* adults were also taxonomically richer than *C. prolifera* adults (*q* = 4.013; *P* < 0.05). The two‐way ANOVA for the inverse Simpson diversity index detected significantly different microbial communities between species (*F*_1,14_ = 13.992; *P* = 0.002) and developmental stage (*F*_1,14_ = 13.599; *P* = 0.002). However, *C. prolifera* developmental stages were not significantly different (*q* = 1.702; *P* = 0.249), and the diversity of microbial communities in larvae from either species were not significantly different (*q* = 1.658; *P* = 0.261). A two‐way ANOVA for evenness found significant differences between species (*F*_1,14_ = 19.063; *P* < 0.001), but not developmental stage (*F*_1,14_ < 0.001; *P* = 0.995), and no interaction was detected (*F*_1,14_ = 0.023; *P* = 0.881).

**Table 1:** Diversity estimators for microbial communities associated with seawater, and mothers and larvae of Clathria prolifera and *Halichondria bowerbanki*.

Source	OTU richness	Inverse Simpson's diversity	Simpson's evenness
Seawater	1359 (37.85)	24.34 (1.40)	0.018 (0.0011)
*Clathria prolifera*			
Mother	1016 (48.02)	3.15 (0.73)	0.003 (0.0006)
Larvae	582 (19.78)	1.84 (0.42)	0.003 (0.0006)
*Halichondria bowerbanki*			
Mother	1283 (135.42)	7.89 (1.43)	0.006 (0.0009)
Larvae	535 (15.00)	3.19 (0.48)	0.006 (0.0009)

All values represent means (±SE).

### *OTU‐level analysis*

**Figure 3:**
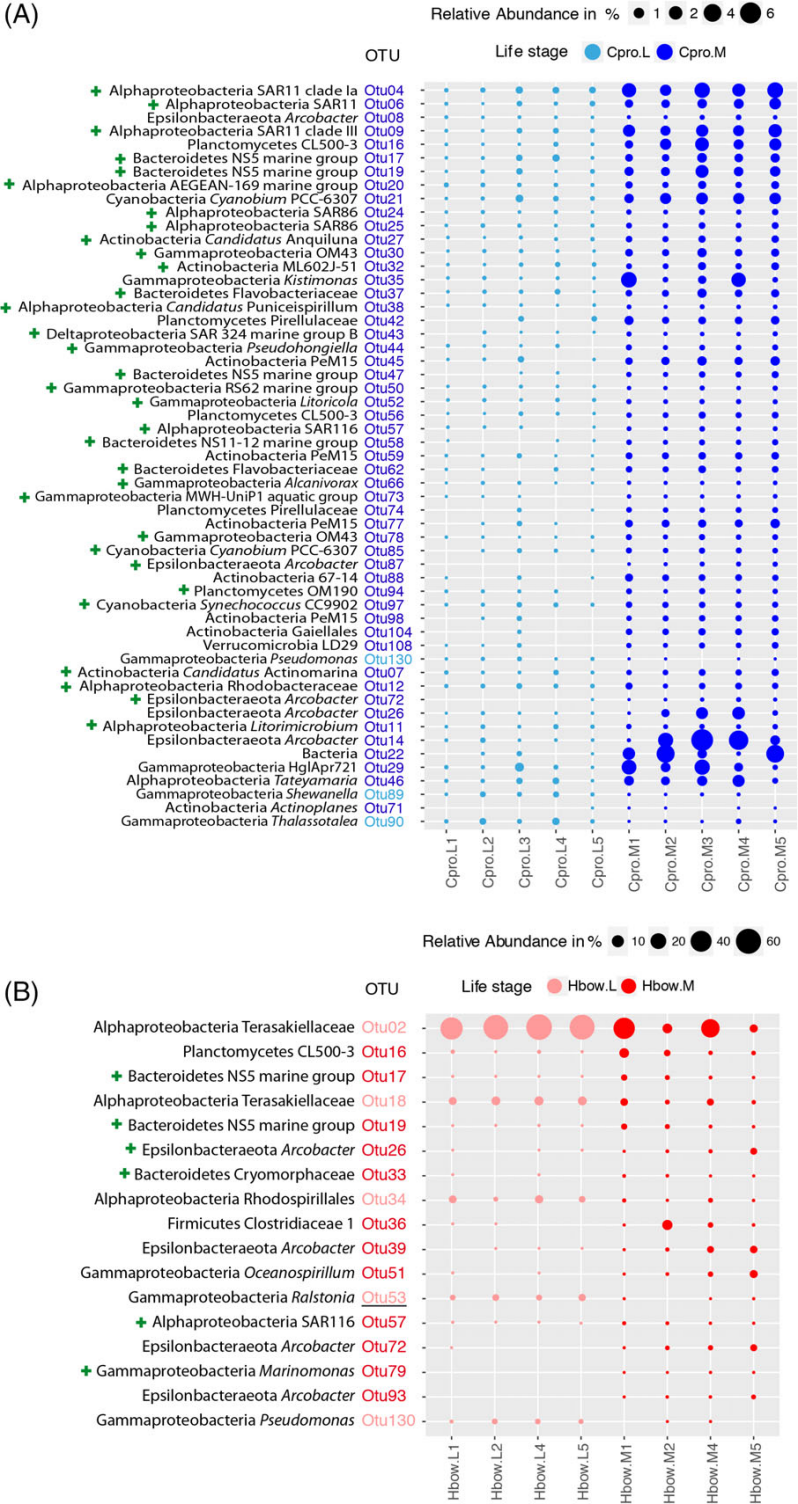
Bubble chart of significantly different OTU abundance between life stages of *Clathria prolifera* (A) and *Halichondria bowerbanki* (B).OTU relative abundances are represented by the size of the bubbles (key on the top of each chart; notice the different scale between A and B). The smallest taxonomical level for each OTU is also shown. *Clathria prolifera* larvae (light blue), *C. prolifera* mothers (blue), *Halichondria bowerbanki* larvae (pink), *H. bowerbanki* mothers (red). The colour of the OTU represents in which sample group (between larvae and adults) is enriched. We also show with a green cross those OTUs enriched in seawater. Underlined OTUs represents those exclusively found in sponge samples. Constructed in R software (version 3.4.3).

**Figure 4:**
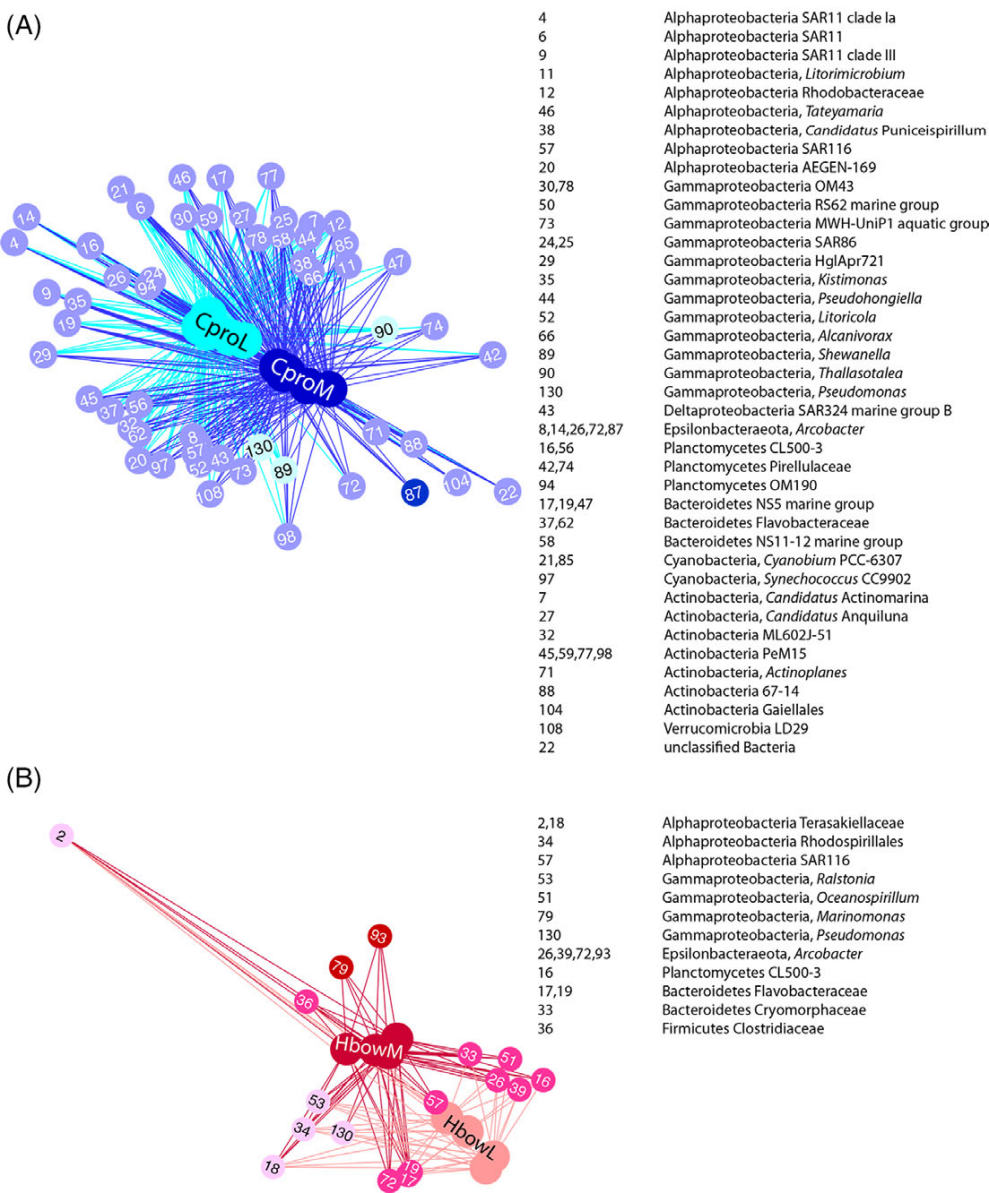
Networks of significantly different abundant OTUs comparing (A) *Clathria prolifera* larvae (CproL) and mothers (CproM) and (B) *Halichondria bowerbanki* larvae (HbowL) and adults (HbowM). We constructed OTU networks with the software Cytoscape v.3.6.1 (Shannon *et al*. 2003) using the edge‐weighted, spring‐embedded algorithm to build a network where nodes (i.e., samples and OTUs) were connected according to a force function (OTU counts). The algorithm sets the positions of the nodes to minimize the sum of forces in the network and establishes a proportional length of the connections to the OTU abundances. The OTU number and its taxonomic assignment of the OTUs are shown. Colour key: dark blue and red OTUs were unique in CproM and HbowM respectively; purple and pink nodes represented shared OTUs but more abundant in CproM and HbowM respectively; light blue and light pink nodes represented shared OTUs but more abundant in CproL and HbowL respectively.

In addition to community‐level metrics of diversity and structure, patterns in the relative abundances of individual symbionts OTUs (based on 97% sequence similarity) across samples were also investigated (Fig. 3; Supporting Information File S1). We found that four OTUs appeared significantly enriched in sponge habitats compared with seawater (17.7% and 0.04% relative abundance respectively), whereas 39 OTUs were significantly more abundant in seawater than in sponges (73.7% and 5.9% relative abundance respectively; Supporting Information Table S1). Fewer than 15% and 10% of the OTUs were vertically‐transmitted to the larvae in *C. prolifera* (*n* = 930 OTUs) and *H. bowerbanki* (*n* = 697 OTUs), respectively, accounting for over 90% of the microbiome in relative abundance. Of those that were vertically‐transmitted, approximately 40% were not detected in seawater (652 OTUs; Supporting Information Table S2). If we analysed life stages, only three OTUs were restricted to adult sponges (Figs 3 and 4), and these were also detected in seawater. Many OTUs (*n* = 61) were found at higher frequencies than expected in adults compared with larval tissue, while a few OTUs (*n* = 8) were at higher proportional representation in larvae compared with mothers than expected by chance (Figs 3 and 4). Comparing both sponge species, some OTUs appeared to be sponge specialists for either *Clathria* (*n* = 6) or *Halichondria* (*n* = 7) in that they were found at significantly higher frequencies in one sponge but not the other (Supporting Information Table S1). Local BLAST searches against the sponge EMP database showed that 90% of the OTUs (*n* = 5963) were found among the sponge microbiome collection with sequence identities over 97% (Supporting Information File S2).

### *Culture‐based approach*

**Figure 5:**
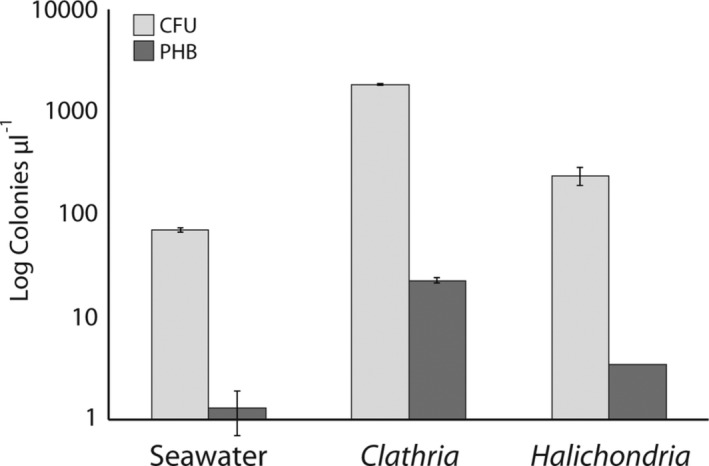
Comparison of colony‐forming units (CFU) and pigmented heterotrophic bacteria (PHB) identified on marine agar plates from seawater and slurries from *Halichondria bowerbanki* and *Clathria prolifera* larvae. Each condition was significantly different in terms of CFU and PHB from the others as indicated by a one‐factor ANOVA (note log scale) performed in R software (version 3.3.3).

**Figure 6:**
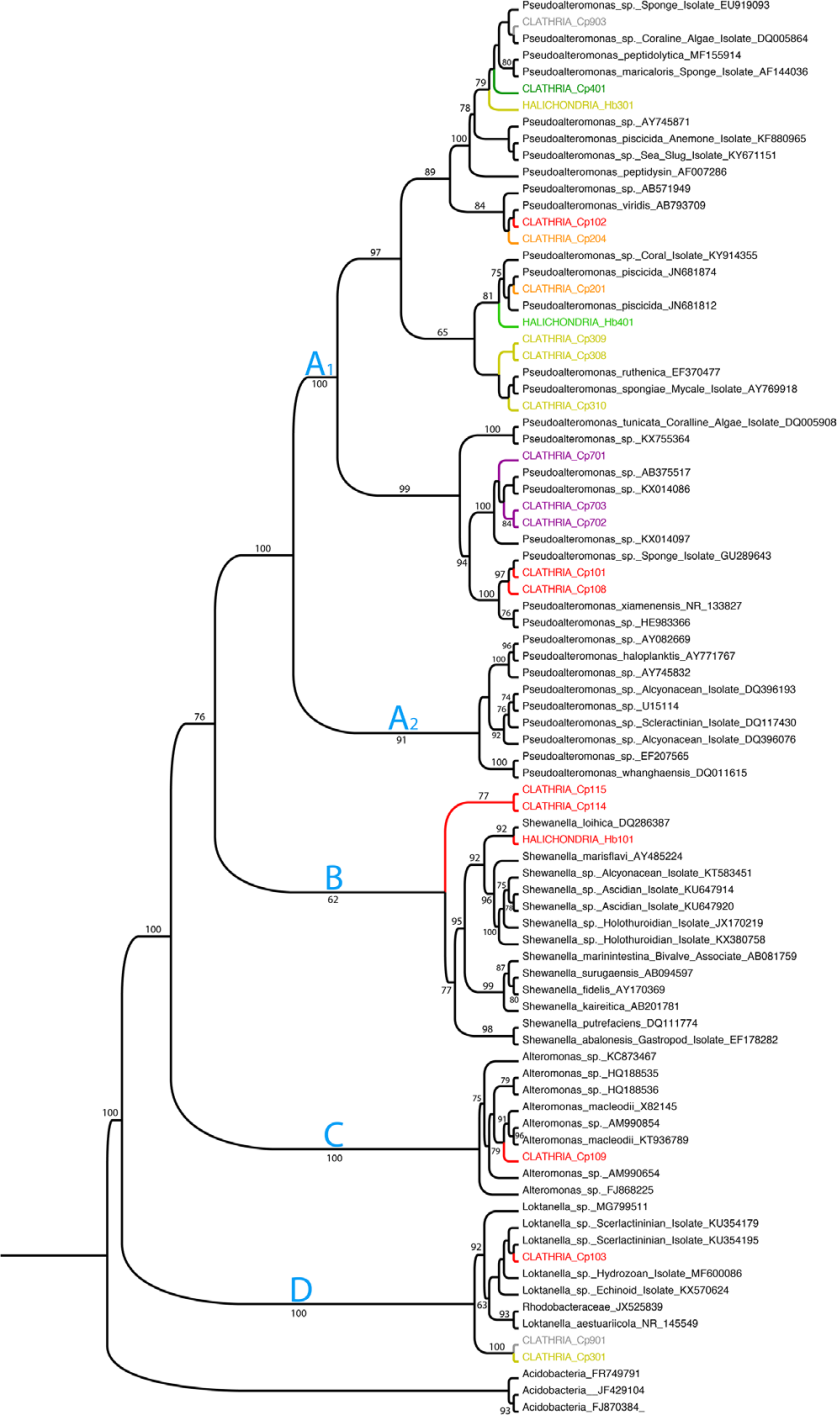
Maximum likelihood phylogeny of selected pigmented heterotrophic bacteria (PHB) derived from *Clathria prolifera* and *Halichondria bowerbanki* larvae. A Tamura‐Nei (TrN) model of substitution (Tamura and Nei 1993) was used in PhyML as implemented in SeaView (Vers. 3.2) to generate trees. We used a Neighbour Joining Tree as a starting tree and the aLRT method to generate support values (Anisimova and Gascuel 2006). Approximate likelihood ratio test support values >60% for particular clades are shown at nodes. Branch colours denote the colour of the PHB colony. Accession numbers for isolates are MH697698–MH697719.

Culture‐based analyses (Supporting Information File S1) revealed a high proportion of pigmented colony‐forming units (CFU), dominated by red and yellow colonies (Supporting Information Fig. S3, Supporting Information Table S3). Significant differences in the density of pigmented heterotrophic bacteria (PHB) were observed with larvae from both sponge species having orders of magnitude more dense bacteria populations than the surrounding seawater (*F*_2,8_ = 18.7; *P* < 0.001; Fig. 5). Furthermore, *C. prolifera* larvae produced more CFU and PHB colonies compared with *Halichondria bowerbanki* (Fig. 5). Local BLAST searches demonstrated that all of the cultured isolates, from which we had a V4 sequence, were found among our microbiome dataset corresponding to rare and abundant OTUs with sequence identities over 99% (Supporting Information Table S3). Sequences from several of the isolated PHB colonies were placed in phylogenetic context (Fig. 6). We focused on the Gammaproteobacteria (Order Alteromonadales: Families Alteromonadaceae, Pseudoalteromonadaceae and Shewanellaceae) and Alphaproteobacteria (Order Rhodobacterales, Family Rhodobacteraceae), which represented the majority of PHB isolated in this study, and are identified by four groups in our phylogeny (Groups A‐D; Fig. 6). Some isolates characterized as *Pseudoalteromonas* and *Phaeobacter* exhibited antibacterial activities (Supporting Information Table S3). Many of the *C. prolifera* isolates had intensely red‐pigmentation (e.g., Cp 101–103, 108, 114, 115; Supporting Information Table S3) and were found in each taxonomic group (Fig. 6). The greatest degree of colony pigmentation was observed among isolates that fell in the *Pseudoalteromonas* lineage, which was split into two main lineages (Groups A_1_ and A_2_). All of our sponge isolates fell in group A_1_, which is a lineage with three other bacteria isolated from sponge sources (Fig. 6). With the exception of the Rhodobacteraceae (Group D), all of the lineages were enriched for bacteria associated with the surfaces of living organisms (as opposed to being derived from abiotic environmental samples). Several lineages that included bacteria isolated from *C. prolifera* and *H. bowerbanki* (e.g., Cp 101 and Cp108, Cp401 and Hb301 and with weaker support, Cp308‐310) fell in lineages that included other sponge isolates (Fig. 6).

## Discussion

As bacteriotrophic predators, sponges would seem unlikely microbial hosts, yet bacteria are ubiquitous sponge symbionts and persist throughout the mesohyl in long‐term relationships. Mechanisms that permit long‐term stability of sponge: microbial partnerships across generations remain poorly understood (Hill and Sacristán‐Soriano 2017). Two important gaps exist in our understanding of sponge symbioses: transmission dynamics and symbiont function (Hill and Sacristán‐Soriano 2017), with the latter hampered by the challenges of cultivating many of the microbes harboured by sponges. We characterized microbial communities in adults and larvae from two pigmented, viviparous, LMA sponges (*Clathria prolifera* and *Halichondria bowerbanki*). Each sponge species harboured unique microbial communities distinct from seawater communities. *Clathria prolifera* microbial communities were dominated by a single microbe belonging to Betaproteobacteriales. *Halichondria bowerbanki* microbial communities were also dominated by a small number of microbes (proteobacteria belonging to the Terasakiellaceae and Vibrionaceae families). The structure of microbial communities in both species resemble other LMA sponges in that they have low diversity communities dominated by one or a few types of bacteria (Lemoine *et al*. 2007; Giles *et al*. 2013; Poppell *et al*. 2014; Moitinho‐Silva *et al*. 2017b). Obvious differences existed in alpha diversity between *H. bowerbanki* and *C. prolifera* larvae with the former harbouring more diverse bacterial taxa. The microbiome found in *C. prolifera* was generally more stable across and within generations than the one found in *H. bowerbanki*. However, consideration of symbiont community composition at a fine taxonomic scale (i.e., individual OTUs defined at 97% sequence similarity) revealed species‐specific microbiome composition of adult and larval tissue. The culturable component of microbial communities found in both sponge species was enriched for pigmented heterotrophic bacteria compared with seawater.

Our results compare favourably to those of Fieth *et al*. (2016) who found ontogenetic shifts in microbiomes for the LMA sponge *Amphimedon queenslandica*, but also evidence of direct transmission. The differences we observed between *C. prolifera*, which had fewer between‐generation differences in the microbiome, and *H. bowerbanki*, which had greater variability among individuals (see also Weigel and Erwin 2016) and generations, indicates that there may be important species‐specificity in these ontogenetic patterns. As in our study, they found that the largest proportion of microbiome variation can be explained by host species. Despite the ubiquity of sponge microbial partners [i.e., 90% of the microbiomes from this study matched at a 3% sequence divergence with those from other sponge species (Moitinho‐Silva *et al*. 2017a)], a particular microbial combination would determine the microbiome of a sponge (Erwin *et al*. 2012b).

Whether the host or the symbionts (or some combination of both) influence ecological structure of microbiomes is unknown, but it is clear that microbial communities found in the earliest developmental stages of the host comprise a subset of the microbial species present in adults. The ultimate composition of sponge symbiont communities may be host‐mediated (e.g. symbiont selection). Enticknap *et al*. (2006) used a culture‐based approach to study an α‐proteobacterium isolated from eight sponge species from the Caribbean and Indo‐Pacific and demonstrated that larvae from *Mycale laxissima* harboured large populations of this symbiont, indicating active transmission mechanisms that generate non‐random patterns of association. It is possible that a process analogous to the winnowing that happens in systems like the *Euprymna*:*Vibrio* light organ symbiosis (Nyholm and McFall‐Ngai 2004) also occurs in sponges. The selection of symbionts may take place from the mesohyl of the sponge acting as environmental pool. Symbiont provisioning to eggs, embryos and developing larvae has been observed in many sponges (e.g., Kaye 1991; Usher *et al*. 2001, 2005; Ereskovsky *et al*. 2005; Maldonado 2007; Schmitt *et al*. 2007; De Caralt *et al*. 2007) and the patterns observed in *C. prolifera* and *H. bowerbanki* may result from selective passage of symbionts by adults. For example, Kaye (1991) observed active transmission of bacteria from maternal cells to embryos through ‘umbilici’, and these symbionts were later found in the low‐density, central region of cyto‐differentiated parenchymella larvae. An alternative to microbe provisioning by adults could be a selective phagocytosis by larvae as phagocytosis of bacteria in larvae transmitted from mother tissue has also been observed (De Caralt *et al*. 2007). Either of these processes, or many other cellular processes, could favour or inhibit transmission of particular microbes.

The accumulation of diverse bacterial lineages in adult sponges may also be symbiont‐mediated (e.g., host invasion). Several authors have proposed molecular mimicry as a strategy for symbionts to avoid detection by the host (Schwarz 2008; Hill and Hill 2012; Hill 2014), a process that may contribute to the observed ontogenetic changes in symbiont communities (from larvae to adult). For example, eukaryotic‐like, ankyrin‐repeat proteins that modulated the phagocytosis behaviour of amoeba have been found in an uncultured γ‐proteobacterial sponge symbiont (Nguyen *et al*. 2014). Nguyen *et al*. (2014) suggested that this might be an escape mechanism by which potential symbionts could make their way into the mesohyl of the sponge after phagocytosis. Based on the results presented herein, we hypothesize that microbiome diversity in adult sponges is primarily symbiont‐mediated (i.e., bacteria invade host tissues from environmental sources), and the larval microbiome is a product of host‐mediated processes at early stages where hosts exert control over symbiont passage to larvae.

Culture‐based approaches have a rich history given that the first attempts to explore sponge microbial communities involved some level of culturing (Wilkinson 1978a,b, 1981; see also Esteves *et al*. 2016). While it has long been recognized that culture‐based approaches miss large percentages of the microbiome (i.e., ‘the great plate count anomaly’ Staley and Konopka 1985), combining molecular‐ and culture‐based approaches offers the potential to relate bacterial and host phenotypes. For example, comparison of bacterial carotenoid profiles to host carotenoid profiles is possible once culturing is possible, and this is a future goal of work with these sponges. Several pigmented bacterial isolates from *Clathria prolifera* (Cp102, Cp204, Cp401, Cp903) and another from *Halichondria bowerbanki* (Hb301) occurred in a lineage that includes two other sponge isolates: *Pseudoalteromonas maricarolis,* isolated from *Fascaplysinopsis reticulata* collected from the Great Barrier Reef (AF144036, Fig. 6; Ivanova *et al*. 2002), and *Pseudoalteromonas* sp. (EU919093), isolated from the invasive sponge *Mycale armata* from Hawaii. The A_1_ group contains a third bacterium (a pale orange *P. spongiae*) isolated from the poecilosclerid *Mycale adhaerens* in Hong Kong (AY769918 in Fig. 6; Lau *et al*. 2005), and three *C. prolifera* PHB were found in this clade (Cp 308–310). Thus, the A_1_ lineage appears to be a rich source of sponge‐isolated bacteria with intriguing phenotypic characteristics including antimicrobial and antifouling activities (Bowman 2007); indeed, nearly half of all the cultured bacteria we examined were found in this lineage.

The pathways that allow persistent partnerships to form between symbiotic bacteria and sponge hosts remain obscure (e.g., Wehrl *et al*. 2007), and the forces that shape ecological community structure of bacterial communities in these symbioses is poorly understood. By studying microbiomes in larvae and adult tissues, we can begin to assess how host‐mediated and symbiont‐mediated processes influence these communities. Indeed, such studies will help us discern the evolutionary forces that mediate host:symbiont conflicts of interest (e.g., Hartmann *et al*. 2017). Through the comparison of two viviparous LMA sponges from the same habitat, our study revealed species‐specific microbiome composition of adult and larval tissue with greater microbial variability among individuals and generations in *Halichondria* compared with a more stable and less diverse microbial community in *Clathria*. We identified a mixed mode of transmission (vertical and horizontal) of microbes between generations in both sponges that might differ between species. We also identified culturable bacteria that offer important opportunities for future experimentation.

## Supporting information


**Figure S1.** Rarefaction curves present the relationship between the sampling effort and the OTU richness in *Clathria prolifera* larvae (CproL) and mothers (CproM), *Halichondria bowerbanki* larvae (HbowL) and adults (HbowM), and ambient seawater (cH2O).

**Figure S2.** Venn diagram showing the unique and shared OTUs among sources defined at distance of 0.03 (i.e., 97% similarity). *Clathria prolifera* (Cpro), *Halichondria bowerbanki* (Hbow), ambient seawater (SW).

**Figure S3.** Culturable microbes isolated from sponge larvae. A. Pigmented CFUs from *Clathria prolifera* larvae (left) compared to ambient seawater CFUs. B. Pigmented CFUs from *Halichondria bowerbanki*.

**Table S1.** Significantly different abundant OTUs in pairwise comparisons among sources according to the false discovery rate (FDR) probability. Mean sequence count of the corresponding source is provided with coloured values representing higher counts. The taxonomy affiliation of each OTU is also shown. Sources: sponges, seawater, *Clathria*, *Halichondria. Clathria* larvae, *Clathria* adults, *Halichondria* larvae, *Halichondria* adults)

**Table S2.** OTUs and their taxonomic affiliation that were solely present in *Clathria* or *Halichondria* (larvae and adults; 1) or in both species (2) but not in seawater.

**Table S3.** Phenotypic features, phylogenetic affiliation, and best hit from local blast searches in the microbiome dataset of selected pigmented heterotrophic bacteria isolated from *Halichondria bowerbanki* (Hb) and *Clathria prolifera* (Cp) larvae. Cell characteristics reported as mean cell size is reported as length x width in μm. Shape, arrangement, and motility of cells were determined by wet‐mount microscopy of cells grown in marine broth. Qualitative assessment of pigment production was also done in marine broth. Gram status was determined using the Gram stain and KOH test (Whitman and MacNair 2004). Antimicrobial activities of heterotrophic bacteria isolated from sponge larvae were assessed by disk diffusion assay on marine agar against *Escherichia coli* (Ec), *Vibrio fischeri* (Vf), *Bacillus subtilis* (Bs), and *Staphylococcus aureus* (Sa). Test bacterial strains were grown in Luria Broth overnight at 37°
C and adjusted to an OD600 of 0.5. Bacterial isolates derived from sponge larvae were grown in Difco® Marine Broth at 30°C for 2–4 days. Sterile filter disks (6 mm diameter) were treated with 10–30 μl of each culture or filter sterilized culture supernatant. Disks were placed on either Difco® Tryptic Soy Agar (TSA) or Marine Agar pre‐streaked with the test bacterial strain. Zones of inhibition were recorded after incubation at 30°C for 2 day and are reported in mm; − indicates no growth inhibition; ND = not determined.

**File S1.** Detailed methodology.

**File S2.** Local blast results of OTUs from *Clathria prolifera* and *Halichondria bowerbanki* against the sponge EMP project database.
