# Supplementary figures and images for: Ontogeny of symbiont community structure in two carotenoid‐rich, viviparous marine sponges: comparison of microbiomes and analysis of culturable pigmented heterotrophic bacteria
Source: Environ Microbiol Rep. 2019 Mar 6;11(2):249–61. doi: 10.1111/1758-2229.12739 (PMC6850349; doi:10.1111/1758-2229.12739)

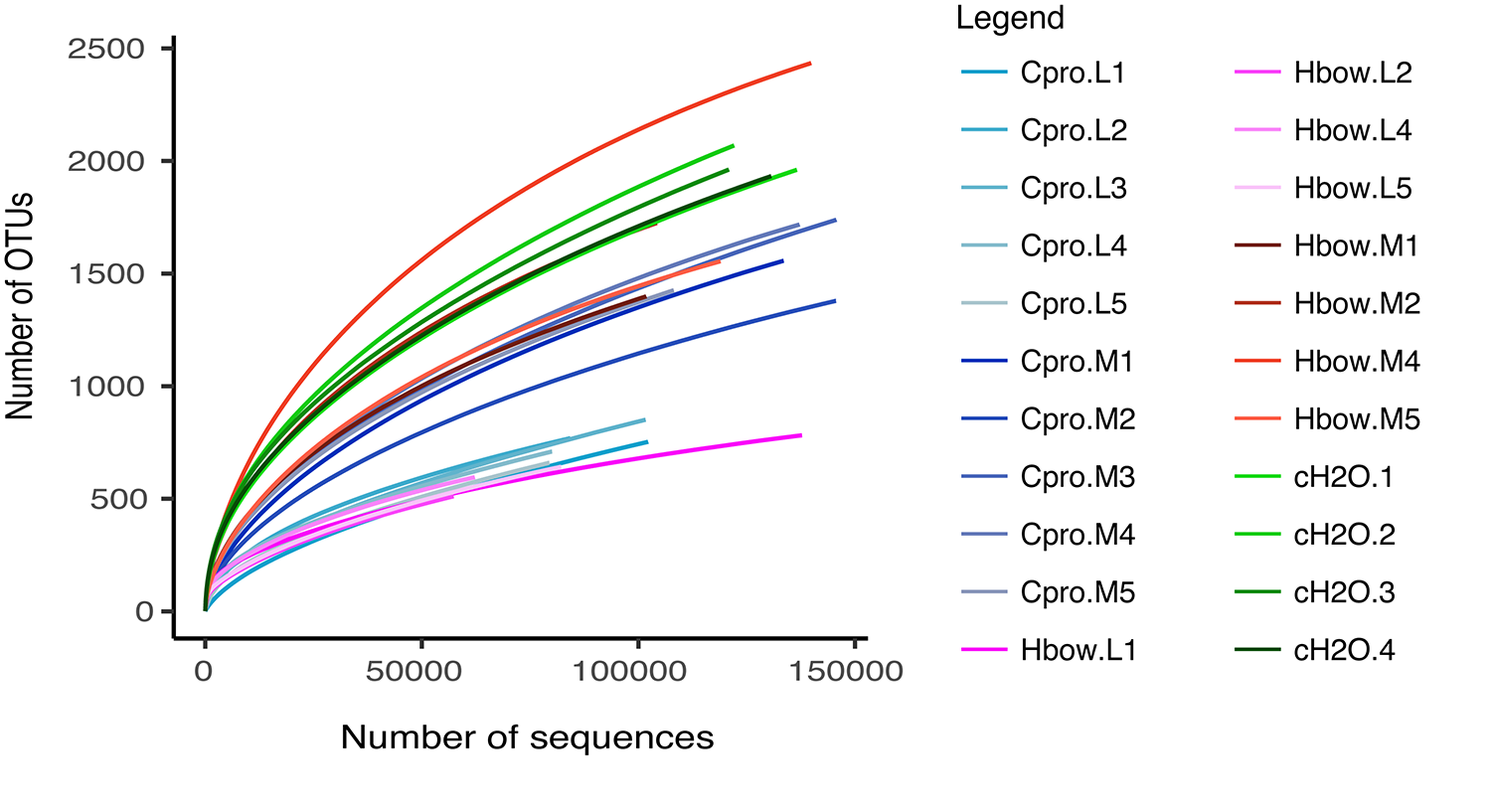

Supplement: Supplementary file 1 — Figure S1. Rarefaction curves present the relationship between the sampling effort and the OTU richness in Clathria prolifera larvae (CproL) and mothers (CproM), Halichondria bowerbanki larvae (HbowL) and adults (HbowM), and ambient seawater (cH2O). [file EMI4-11-249-s001.tif]

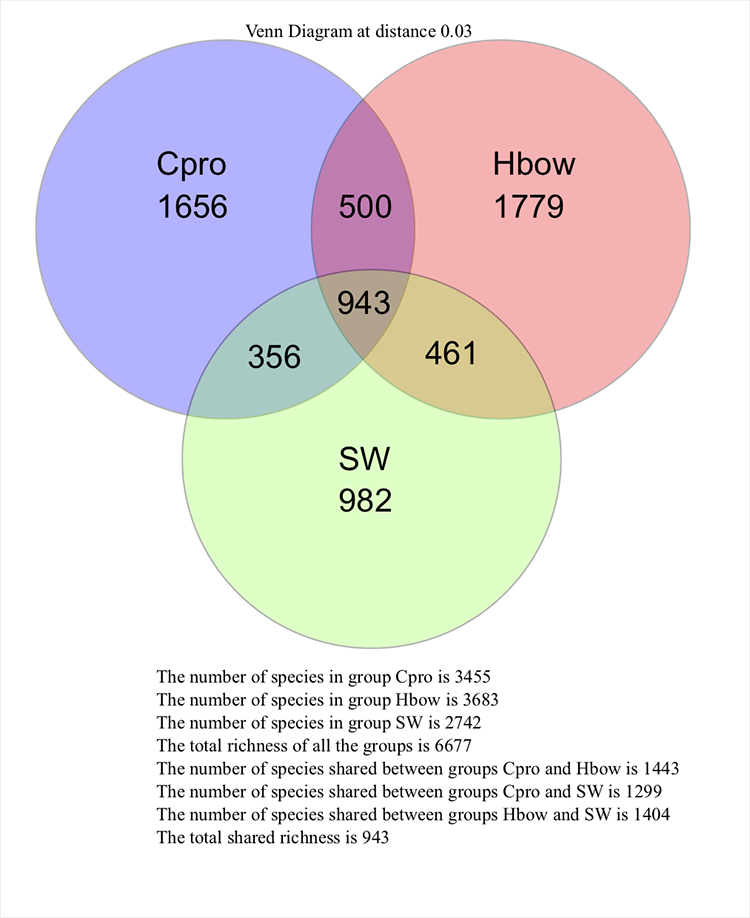

Supplement: Supplementary file 2 — Figure S2. Venn diagram showing the unique and shared OTUs among sources defined at distance of 0.03 (i.e., 97% similarity). Clathria prolifera (Cpro), Halichondria bowerbanki (Hbow), ambient seawater (SW). [file EMI4-11-249-s002.tiff]

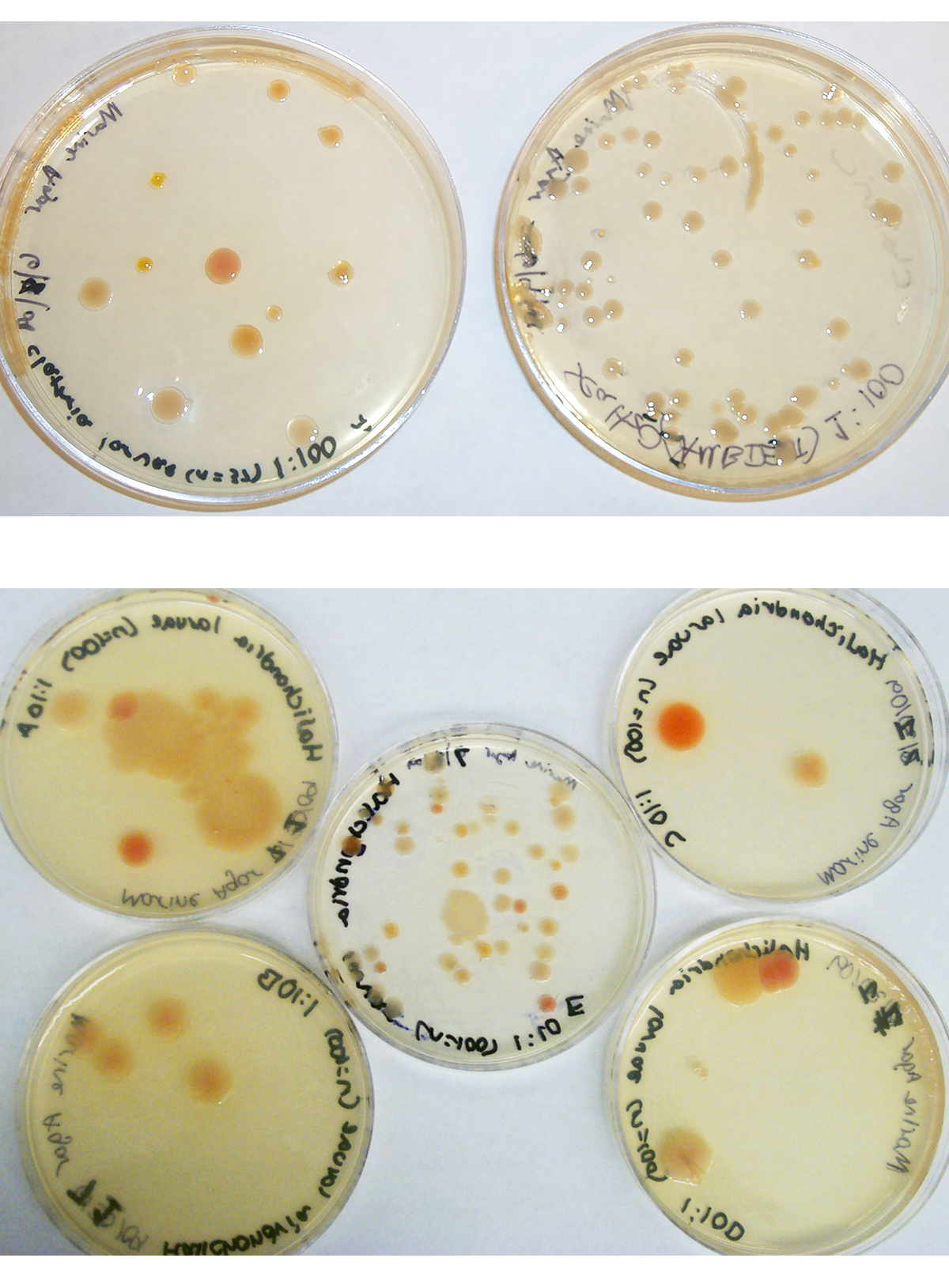

Supplement: Supplementary file 3 — Figure S3. Culturable microbes isolated from sponge larvae. A. Pigmented CFUs from Clathria prolifera larvae (left) compared to ambient seawater CFUs. B. Pigmented CFUs from Halichondria bowerbanki. [file EMI4-11-249-s003.tif]
