# Supplementary material for: Ontogeny of symbiont community structure in two carotenoid‐rich, viviparous marine sponges: comparison of microbiomes and analysis of culturable pigmented heterotrophic bacteria
Source: Environ Microbiol Rep. 2019 Mar 6;11(2):249–61. doi: 10.1111/1758-2229.12739 (PMC6850349; doi:10.1111/1758-2229.12739)
Supplement: Supplementary file 6 — Table S3. Phenotypic features, phylogenetic affiliation, and best hit from local blast searches in the microbiome dataset of selected pigmented heterotrophic bacteria isolated from Halichondria bowerbanki (Hb) and Clathria prolifera (Cp) larvae. Cell characteristics reported as mean cell size is reported as length x width in μm. Shape, arrangement, and motility of cells were determined by wet‐mount microscopy of cells grown in marine broth. Qualitative assessment of pigment production was also done in marine broth. Gram status was determined using the Gram stain and KOH test (Whitman and MacNair 2004). Antimicrobial activities of heterotrophic bacteria isolated from sponge larvae were assessed by disk diffusion assay on marine agar against Escherichia coli (Ec), Vibrio fischeri (Vf), Bacillus subtilis (Bs), and Staphylococcus aureus (Sa). Test bacterial strains were grown in Luria Broth overnight at 37° C and adjusted to an OD600 of 0.5. Bacterial isolates derived from sponge larvae were grown in Difco® Marine Broth at 30°C for 2–4 days. Sterile filter disks (6 mm diameter) were treated with 10–30 μl of each culture or filter sterilized culture supernatant. Disks were placed on either Difco® Tryptic Soy Agar (TSA) or Marine Agar pre‐streaked with the test bacterial strain. Zones of inhibition were recorded after incubation at 30°C for 2 day and are reported in mm; − indicates no growth inhibition; ND = not determined. [file EMI4-11-249-s006.docx]

Supplemental Table 2. Phenotypic feature, phylogenetic affiliation and best hit from local blast searches in the microbiome dataset of selected pigmented heterotrophic bacteria isolated from *Halichondria bowerbanki* (Hb) and *Clathria prolifera* (Cp) larvae. Cell characteristics reported as mean cell size is reported as length x width in µm. Shape, arrangement, and motility of cells were determined by wet-mount microscopy of cells grown in marine broth. Qualitative assessment of pigment production was also done in marine broth. Gram status was determined using the Gram stain and KOH test (Whitman and MacNair 2004). Antimicrobial activities of heterotrophic bacteria isolated from sponge larvae were assessed by disk diffusion assay on marine agar against *Escherichia coli* (Ec), *Vibrio fischeri* (Vf), *Bacillus subtilis* (Bs), and *Staphylococcus aureus* (Sa). Test bacterial strains were grown in Luria Broth overnight at 37˚C and adjusted to an OD_600_ of 0.5. Bacterial isolates derived from sponge larvae were grown in Difco^®^ Marine Broth at 30˚C for 2-4 days. Sterile filter disks (6 mm diameter) were treated with 10-30 µl of each culture or filter sterilized culture supernatant. Disks were placed on either Difco^®^ Tryptic Soy Agar (TSA) or Marine Agar pre-streaked with the test bacterial strain. Zones of inhibition after incubation at 30˚C for 2 day are reported in mm; - indicates no growth inhibition; ND = not determined.

Supplemental Table 2 (cont)

|  |  |  |  |  |  |  | Antibacterial Assays | | | |
| --- | --- | --- | --- | --- | --- | --- | --- | --- | --- | --- |
| Isolate | Cell characteristics | Pigment | Motility | Phylogenetic affiliation | Best hit microbiome | Gram | Ec | Vf | Bs | Sa |
| Hb101 | 1.5 X 0.5, rod, single & pairs | Light orange | + | *Shewanellaceae* | ** | - | - | ND | - | - |
| Hb301 | 2.5 X 0.5, rod, mostly single | Orange | + | *Pseudoalteromonadaceae* | OTU5 | - | - | 14 | 14 | 10 |
| Hb302 | 2-1.5 X <0.5, rod, single | Yellow | -* | *Flavobacteriaceae* | ** | - | - | ND | - | - |
| Cp101 | 2-3 X 0.5, rod, single & pairs | Red/metallic | + | *Pseudoalteromonadaceae* | OTU359 | - | - | ND | - | - |
| Cp102 | rod | Red | + | *Pseudoalteromonadaceae* | OTU5 | - | - | ND | - | - |
| Cp103 | 1 X 1, rod, single/pairs/rosette | Pink | + | Rhodobacteraceae | ** | - | - | ND | - | - |
| Cp108 | 2 X 1, rod, single/pairs/short chains | Red | + | *Pseudoalteromonadaceae* | OTU359 | - | - | ND | - | - |
| Cp201 | 1.5 X 1, rod, single & pairs | Orange | + | *Pseudoalteromonadaceae* | OTU5 | - | - | ND | 12 | 10 |
| Cp301 | 2 X 1, rod, single/pairs/rosette | Brown | + | Rhodobacteraceae | ** | - | - | 20 | 12 | - |
| Cp401 | 2 X 1, rod, single & pairs | Yellow | + | *Pseudoalteromonadaceae* | OTU5 | - | - | ND | 8 | - |
| Cp901 | 1.5 X 1, rod, rosettes & large clusters | Black | + | Rhodobacteraceae | ** | - | - | ND | - | - |

* The *Flavobacteriaceae* isolate (*Tenacibaculum)* did not exhibit motility on a wet mount, but is known to exhibit gliding motility on solid surfaces (Suzuki et al., 2001).

**16S rDNA V4 region not available.
