# Supplementary material for: Ontogeny of symbiont community structure in two carotenoid‐rich, viviparous marine sponges: comparison of microbiomes and analysis of culturable pigmented heterotrophic bacteria
Source: Environ Microbiol Rep. 2019 Mar 6;11(2):249–61. doi: 10.1111/1758-2229.12739 (PMC6850349; doi:10.1111/1758-2229.12739)
Supplement: Supplementary file 7 — File S1. Detailed methodology. [file EMI4-11-249-s007.docx]

**Methods and materials**

*Sample collection*

Sponges were transported to the lab within 2 hours of collection where they were placed into separate illuminated tanks with aerated 0.22 µm-filtered seawater from the field. Salinity was ≈ 17 psu at the time of collection. Replicate seawater samples (n = 4, l l samples) were aseptically collected adjacent to the sampled sponges in the field.

*Microbiome analysis*

Larvae from a single tank (i.e., from a single mother) were collected as they were released, and all larvae were collected within 4 hours of mother sponges arriving at the lab. Pooled larvae (n = 50 larvae per 1.5 ml tube) were rinsed at least 5 times in 250-500 µl of sterile 0.22 µm-filtered seawater (SFS). To ensure maximal removal of environmental water, tubes were briefly centrifuged to collect larvae at the bottom of the tube, and nearly all water was removed before the next rinse. After the final rinse, larvae were pelleted by centrifugation at 16,000 rcf, and all residual seawater was removed. Larval populations from 5 *C. prolifera* mothers, and 4 *H. bowerbanki* individuals were used for subsequent microbiome analysis. A small piece of maternal adult tissue was collected from each individual after larval collection. Seawater from the field (n =4 samples) was passed through polycarbonate 5 µm and 0.22 µm filters (sequentially; MilliporeSigma, Burlington, MA, USA), and the contents on the 0.22 µm filters were used to examine the ambient bacterioplankton communities. All filters, sponge and larval samples were kept frozen until processed. DNA was extracted using the DNeasy PowerSoil kit (QIAGEN, Germantown, MD, USA) following standard protocols of the Earth Microbiome Project (http://press.igsb.anl.gov/earthmicrobiome/emp-standard-protocols/dna-extraction-protocol/). DNA extracts were submitted to Molecular Research LP ([www.mrdnalab.com](https://www.dropbox.com/referrer_cleansing_redirect?hmac=k03wxuc4EuLklRhD3TJLS4LOPqeqZ%2FwqnSrEOT%2F%2FKg0%3D&url=http%3A%2F%2Fwww.mrdnalab.com), Shallowater, TX, USA) for amplification, library construction and multiplexed sequencing of partial (V4) 16S rRNA gene sequences on an Illumina MiSeq platform. The HotStarTaq Plus Master Mix kit (Qiagen) was used for PCR amplifications using DNA extracts as templates with universal bacterial/archaeal forward and reverse primers 515fb (5'-GTGYCAGCMGCCGCGGTAA-3') and 806rb (5'-GGACTACNVGGGTWTCTAAT-3'), respectively (Caporaso et al. 2011; Apprill et al. 2015). To barcode samples, a multiplex identifier barcode was attached to the forward primer. The thermocycler profile consisted of an initial denaturation step at 94 °C for 3 min; 28 cycles of 94°C for 30s, 53°C for 40s, and 72°C for 1 min with a final elongation step at 72°C for 5 min. Equimolar concentrations of samples were pooled and purified using Agencourt Ampure XP beads (Beckman Coulter) to prepare DNA library by following Illumina TruSeq DNA library preparation protocol. Sequencing was then performed according to manufacturer’s guidelines on an Illumina MiSeq. Illumina sequence data were deposited in NCBI SRA under the accession number SRP149479.

As described previously (Thomas et al. 2016), Illumina sequence reads were processed in mothur v1.39.5 (Schloss et al. 2009). Briefly, raw reads were demultiplexed, forward and reverse reads were then joined and sequences <200bp and with ambiguous base calls were removed. Sequences were aligned to the SILVA database (release 128, non-redundant, mothur-formatted), trimmed to the V4 region, and screened for chimeras and errors. A naïve Bayesian classifier and Greengenes taxonomy (August 2013 release, mothur-formatted) was used to aid in the removal of non-target sequences (e.g., chloroplasts, mitochondria). We used the SILVA database (release 132, non-redundant, mothur-formatted) for final taxonomic assignment. The resulting high-quality sequences were clustered into operational taxonomic units (OTUs) defined by clustering at 3% divergence and singletons were removed. We used rarefaction curves (R software version 3.4.3) to plot the OTUs observed as a function of sequencing depth. To avoid artifacts of varied sampling depth on subsequent diversity calculations, each sequence dataset was subsampled to the lowest read count. To place the determined OTUs into a greater context, these were compared to the database of the sponge EMP project (Moitinho-Silva et al. 2017a) using local BLAST searches (NCBI-BLAST-2.7.1+).

*Community-level analysis*

Significant differences among sponge species and ambient seawater were assessed using a one-way permutational multivariate analysis of variance (PERMANOVA), with the factor source (*C. prolifera* vs. *H.bowerbanki* vs. seawater). Significant differences between sponge species and developmental stages were assessed using a two-way PERMANOVA, with the factors source (*C. prolifera* vs. *H.bowerbanki*), life stage (larvae vs. adult) and an interaction term. Pairwise comparisons were subsequently conducted for all significant PERMANOVA results. Permutational multivariate analysis of dispersion (PERMDISP) was used to detect differences in homogeneity (dispersion) among groups for all significant PERMANOVA outcomes. All multivariate statistics were performed using Primer 7.0.13 and PERMANOVA+ (Plymouth Marine Laboratory, United Kingdom).

We calculated three indices of alpha diversity in mothur v1.39.5 (Schloss et al. 2009) to evaluate community richness and evenness: observed OTU richness, the Simpson index of evenness and the inverse of Simpson index of diversity. Two-way analysis of variance (ANOVA) was used to detect differences in diversity metrics by the factors source (sponge species), life stage (larvae vs. adult) and an interaction term, followed by pairwise comparisons for any significant factor. All data that did not meet the statistical assumptions was transformed accordingly. The univariate statistics were performed using Sigmaplot v11.

*OTU-level analysis*

We analyzed the dataset for patterns in relative abundances of particular OTUs within categories (e.g., sponge vs. seawater, mother vs. larvae). For this purpose, we removed from the dataset rare OTUs (<0.1% relative abundance) and OTUs with a low incidence across samples (detected in <2 samples). We used the Mann-Whitney-U test (or Wilcoxon rank sum test) with FDR p-value correction to identify significantly different patterns in OTU relative abundance among hosts and life stages using QIIME (Caporaso et al. 2010).

*Culturing pigmented heterotrophic bacteria*

To compare bacterial densities found in ambient seawater to those found in *C. prolifera* and *H. bowerbanki* larvae, we used a modified methodology from Du et al. (2006). We first volumetrically standardized the amount of sample used to inoculate cultures. We estimated the volumes of individual sponge larvae by measuring longitudinal and latitudinal diameters using ImageJ (Schneider et al. 2012) from digital images of free-swimming *C. prolifera* (n = 239) and *H. bowerbanki* (n = 209) larvae. Volume was calculated using the equation for a prolate spheroid (1). *Clathria* larvae were found to be significantly larger (mean = 6.94 nl) than *Halichondria* larvae (mean = 2.07 nl), but much more variable in size (sd = 2.67 vs. 0.72, respectively).

$\left( 1 \right) V=\frac{4}{3}\pi a^{2}b$; where $a$ and $b$ are the equatorial and polar radius, respectively.

Three volumetrically equivalent (≈ 1 µl each) larval samples from each species (n ≈ 145 larvae from *C. prolifera*; n ≈ 480 larvae from *H. bowerbanki*) were re-suspended in separate tubes to 100 µl with SFS. Larvae were spun into a pellet (≈8000 rcf, 10 sec), and excess water was removed. This process was repeated 12 times. The final spin was done at maximum rcf (>13000 rcf) for 1 min to completely pellet the larvae, and all remaining water was removed. The pellet was crushed with a nylon pestle in 50 µl of SFS, and the resulting slurry was diluted to 100 µl with SFS. The larval solution was mixed thoroughly, and serially diluted with SFS (1X, 1:2, 1:10, and 1:100). A 100 µl aliquot of each was then plated on Marine Agar (Difco™ 2216). We established replicate plates (n = 3) for each concentration. We also plated 1 µl of unfiltered field seawater (diluted in SFS) collected near the mother sponges in replicate plates (n = 3 per dilution) using the dilution series described above. We ensured that seawater samples were devoid of sponge larvae. Negative control plates were inoculated with the SFS to verify that it was not a source of any cultured bacteria. Plates were incubated in the dark at room temperature for 4 days before colonies were examined and counted. Once we verified no growth on our negative controls, we compared larval- and seawater-inoculated plates. All colony forming units (CFU) were counted, as were any pigmented heterotrophic bacteria (PHB). From these counts, we estimated the density of bacteria associated with sponge or environmental samples (e.g., number of CFU µl^-1^), and statistical comparisons of densities were performed in R software (version 3.3.3). Pure cultures of the PHB were created after counts were completed by sub-culturing bacterial colonies. Frozen stocks of pure isolates were stored in 20% glycerol and Marine Broth (Zobell 2216) for subsequent phenotypic work.

*Phenotypic work on culture isolates*

We assessed gram-status of PHB via the KOH test (Whitman and MacNair 2004) and Gram stain. We examined the antimicrobial activities of bacterial isolates against Gram-positive bacteria using a disk diffusion assay. Test bacterial strains were grown in Luria Broth overnight at 37˚C and adjusted to an OD_600_ of 0.5. Bacterial isolates derived from sponge larvae were grown in Difco^®^ Marine Broth at 30˚C for 2-4 days. Sterile filter disks (6 mm diameter) were treated with 10-30 µl of each culture or filter sterilized culture supernatant. Disks were placed on either Difco^®^ Tryptic Soy Agar (TSA) or Marine Agar pre-streaked with the test bacterial strain. Plates were incubated at 30˚C for 2 days and the diameters of any zone of inhibition were measured. Finally, motility and morphology of isolates was assessed by wet mount phase contrast microscopy from broth-based cultures.

*Phylogenetic analysis on culture isolates*

We isolated DNA from each bacterial isolate using the DNeasy PowerSoil kit (QIAGEN, Germantown, MD, USA). PCR amplification of 16S rDNA was done using two sets of primers, the 27f and 1492r (for isolates sequenced at Fairfield University; Lane 1991) and the V4 primers (for those analyzed at the University of Richmond; see *Microbiome analysis* section). PCR conditions included 4 min at 94°C; 30 cycles of 30 sec at 94°C, 30 sec at 55°C or 53˚C (respectively), and 45 sec at 72°C. A final elongation step of 2 min at 72°C was included. PCR products were separated on a 1% agarose gel to verify amplification. Amplicons were cleaned using the QIAGEN PCR Clean Up Kit. Isolates examined at Fairfield University were sequenced using a Beckman Coulter CEQ™ DTCS kit with the CEQ8000 DNA Analysis System; all other isolate amplicons were sequenced using SimpleSeq™ services through Eurofins Genomics. Data from 22 isolates were deposited in NCBI under the accession numbers MH697698-MH697719.

Representative sequences with high degree of similarity to the larval PHB isolates were compiled via BLAST (<https://blast.ncbi.nlm.nih.gov>). Subsequent alignment was performed with Muscle (Edgar 2004) implemented in SeaView (Vers. 3.2; Gouy et al. 2010). jModelTest (Posada 2008) suggested a **Tamura-Nei (TrN)** model of substitution (Tamura and Nei 1993), which was used in PhyML as implemented in SeaView to generate trees. We used a Neighbor Joining Tree as a starting tree and the aLRT method to generate support values (Anisimova and Gascuel 2006).

To determine whether the cultured isolates were also recovered by the high-throughput sequencing techniques, we performed a local blast of the isolates that had the V4 region included in their sequence against our 16S microbiome sequencing data (NCBI-BLAST-2.7.1+).

**References**

Apprill, A., McNally, S., Parsons, R., and Weber, L. (2015) Minor revision to V4 region SSU rRNA 806R gene primer greatly increases detection of SAR11 bacterioplankton. Aquat Microb Ecol 75: 129–137.

Caporaso, J.G., Kuczynski, J., Stombaugh, J., Bittinger, K., Bushman, F.D., Costello, E.K., Fierer, N., Gonzalez Pena, A., Goodrich, J.K., Gordon, J.I., Huttley, G.A., Kelley, S.T., Knights, D., Koenig, J.E., Ley, R.E., Lozupone, C.A., McDonald, D., Muegge, B.D., Pirrung, M., Reeder, J., Sevinsky, J.R., Turnbaugh, P.J., Walters, W.A., Widmann, J., Yatsunenko, T., Zaneveld, J., and Knight, R. (2010) QIIME allows analysis of high-throughput community sequencing data. Nature Methods 7: 335-336.

Caporaso, J.G., Lauber, C.L., Walters, W.A., Berg-Lyons, D., Lozupone, C.A., Turnbaugh, P.J., Fierer, N., and Knight, R. (2011) Global patterns of 16S rRNA diversity at a depth of millions of sequences per sample. Proc Natl Acad Sci 108: 4516–4522.

Du, H., Jiao, N., Hu, Y., and Zeng, Y. (2006) Diversity and distribution of pigmented heterotrophic bacteria in marine environments. FEMS Microbiol Ecol 57: 92-105.

Edgar, R.C. (2004) MUSCLE: multiple sequence alignment with high accuracy and high throughput. Nucleic Acids Res 32: 1792–1797.

Gouy, M., Guindon, S., and Gascuel, O. (2010) SeaView Version 4: A multiplatform graphical user interface for sequence alignment and phylogenetic tree building. Mol Biol Evol 27: 221–224.

Lane, D.J. (1991) 16S/23S rRNA sequencing. In: Nucleic acid techniques in bacterial systematics. Stakebrandt, E., and Goodfellow, M. (eds). John Wiley and Sons, New York, NY, pp. 115-175

Moitinho-Silva, L., Nielsen, S., Amir, A., Gonzalez, A., Ackermann, G.L., Cerrano, C., Astudillo-Garcia, C., Easson, C., Sipkema, D., Liu, F., Steinert, G., Kotoulas, G., McCormack, G.P., Feng, G., Bell, J.J., Vicente, J., Björk, J.R., Montoya, J.M., Olson, J.B., Reveillaud, J., Steindler, L., Pineda, M.C., Marra, M.V., Ilan, M., Taylor, M.W., Polymenakou, P., Erwin, P.M., Schupp, P.J., Simister, R.L., Knight, R., Thacker, R.W., Costa, R., Hill, R.T., Lopez-Legentil, S., Dailianis, T., Ravasi, T., Hentschel, U., Li, Z., Webster, N.S., and Thomas, T. (2017a) The sponge microbiome project. GigaScience 6:1-7.

Posada, D. (2008) jModelTest: Phylogenetic model averaging. Mol Biol Evol 25: 1253-1256.

Schloss, P. D., Westcott, S. L., Ryabin, T., Hall, J. R., Hartmann, M., Hollister, E. B., et al. (2009). Introducing mothur: open- source, platform-independent, community-supported software for describing and comparing microbial communities. Appl Environ Microbiol 75, 7537–7541.

Schneider, C.A., Rasband, W.S., and Eliceiri, K.W. (2012) NIH Image to ImageJ: 25 years of image analysis. Nature Methods 9: 671-675.
